# Annual Athlete Turnover of 36% in Olympic Talent Promotion Programs: A Meta-analysis

**DOI:** 10.1186/s40798-026-01050-9

**Published:** 2026-06-30

**Authors:** Arne Güllich, Michael Barth

**Affiliations:** 1 Department of Sports Science, RPTU Kaiserslautern, Erwin-Schrödinger-Straße 57, 67663 Kaiserslautern, Germany; 2 Department of Sport Science, University of Innsbruck, Fürstenweg 185, 6020 Innsbruck, Austria

**Keywords:** Talent identification, Talent development, Talent promotion programs, Fluctuation, Performance, Sustainability, Olympic sports, Meta-analysis

## Abstract

**Background:**

Across the world, many national sport systems make extensive strategic investments in talent promotion programs (TPPs). The most common TPPs include youth sport academies and sport federations’ junior squads. The central idea of TPPs is (1) to select the most promising talents at young ages (around puberty or younger) and (2) to involve them in a long-term continuous nurturing process to facilitate their performance development, eventually leading to increased senior peak performance. This central idea implies that the population of TPP participants is highly stable across age categories. On the other hand, TPPs sometimes replace deselected athletes with new ‘side-entry’ athletes (athletes who enter a TPP after its initial age category). Hence, there is athlete turnover—fluctuation in the TPP population through exits of participants and entries of new athletes. This implies that the TPP population is unstable across age categories. The magnitude of annual athlete turnover is thus indicative of the operating principle of TPPs and whether it corresponds to their central idea.

**Objective:**

This meta-analysis aimed to provide robust and generalizable evidence on the magnitude of annual athlete turnover in TPPs. It also investigated whether athlete turnover varies between youth sport academies vs federations’ junior squads and across ages and sexes.

**Methods:**

A systematic literature search was conducted in March 2025 in Web of Science, PubMed, APA PsycINFO, APA PsycARTICLES, and Google Scholar, complemented by snowball search. We searched for original studies that reported annual athlete turnover within TPPs or data needed to compute annual athlete turnover. The search yielded 37 samples, published 2010–25, including 44,287 athletes from all Olympic sports and 42 countries. For each TPP sample and each season-to-season transition, annual athlete turnover was calculated as (number of entries + number of exits) / 2 / total number of current athletes.

**Results:**

The mean annual athlete turnover within TPPs is 36.3%. It is slightly higher in federations’ junior squads than youth sport academies but does not differ across ages or sexes.

**Discussion:**

Most talent selection decisions are revised within two years and, accordingly, more than half of the TPP population is exchanged every two years. Unlike their original idea, the operating principle of TPPs is characterized by frequent selection, deselection, and replacement of youth athletes, resulting in sizeable athlete turnover. We discuss implications regarding inaccurate talent identification and inefficient TPP nurture.

**Conclusion:**

The operating principle of TPPs can be described as follows: TPPs try out many youth athletes and expand that number through sizeable athlete turnover. Most talent-identified youth athletes are deselected again soon and replaced with others who are then tried out.

**Supplementary Information:**

The online version contains supplementary material available at https://doi.org/10.1186/s40798-026-01050-9.

## Key Points

- The central idea of talent promotion programs (TPPs) is to select the most promising young talents and involve them in a long-term continuous nurturing process to facilitate their performance development, eventually leading to increased senior peak performance.
- Other than their original idea, the operating principle of TPPs is characterized by frequent selection, deselection, and replacement of youth athletes across age categories, resulting in 36.3% annual athlete turnover.
- Most talent selection decisions are considered invalid within two years and are revised, and, accordingly, more than half of the TPP population is exchanged every two years.

**Supplementary Information:**

The online version contains supplementary material available at https://doi.org/10.1186/s40798-026-01050-9.

## Introduction

Many national sport systems around the world have established talent promotion programs (henceforth TPPs) at local, regional, and national levels.^1^ The foundational idea of TPPs is to select the most promising young talents and foster their performance development in a long-term nurturing process until peak performance. In this meta-analysis, we investigate to which extent the operating principle of TPPs corresponds to this foundational idea or is characterized by repeated selection, deselection, and replacement of youth athletes across age categories.

TPPs are considered a critical building block of athletes’ pathway towards athletic excellence and an important ‘tool’ to promote the development of youth athletes. TPPs are therefore a major resource of nations, sport federations, and professional sports clubs in the “sporting arms race” [2], and national and international competition has stimulated extensive strategic investments in TPPs [2–10].

The most common TPPs include youth sport academies^2^ and sport federations’ junior squads (including under-age selection teams) [1, 3, 8, 11–26]. Youth sport academies typically operate year round and provide day-to-day training. Federations’ junior squads usually gather for competitions, training camps, and clinics over several weekends or weeks annually.

The programs provide environments, resources, and interventions to talent-identified youth athletes to foster their performance progress [3, 12, 17, 23, 24, 27–33]. These may include participation in additional, high-level competitions; additional training opportunities; increased training volume; training camps and clinics; training and competing with other athletes who have a similar performance level; educated, high-profile coaches; high-profile facilities; and sportswear and equipment provided to youth athletes. They may also include support staff providing sports medicine, physiotherapy, biomechanical and physiological performance analysis, nutritional counselling, career and lifestyle counselling, psychological support, and academic assistance. Finally, they often include school timetables adjusted to the sport schedule; transportation; residency; and financial funding provided to youth athletes.

TPPs typically seek to select talents at young age—often around puberty or younger—for three major reasons. The first is the widespread belief that beginning to foster the youth athlete’s development through the TPP nurture at a younger age will better facilitate long-term performance development. The second reason is that TPPs seek to secure themselves the talents, before other TPPs and other sports. The final reason is that beginning at a young age enables a long total period of continuous TPP nurture until the anticipated age of peak performance [1, 12, 17, 20, 21, 25, 26, 34–45]. The selection for TPPs is often based on early performance—in competition or in representative tests of relevant motor abilities and skills or both—which is evaluated by objective data or coach assessment or both [12, 17, 21, 46–62].

In summary, the central idea of TPPs is (1) to identify and select the most promising young talents who possess the greatest long-term potential to develop eventual senior elite performance (i.e., in the highest, open-age category, typically in their 20s–30s), and (2) to involve them in a long-term continuous nurturing process that facilitates their performance progress, eventually leading to increased peak performance at senior age. An assumption of this approach is that the talents with the greatest long-term potential are accurately identified and distinguished from those with less potential within the ‘risk population’—the youth athletes considered for potential selection—at a young age. A further assumption is that the environments, resources, and interventions provided to TPP participants are superior in fostering their long-term performance development compared with developmental conditions in general sport clubs or schools outside the TPP system.

**Fig. 1:**
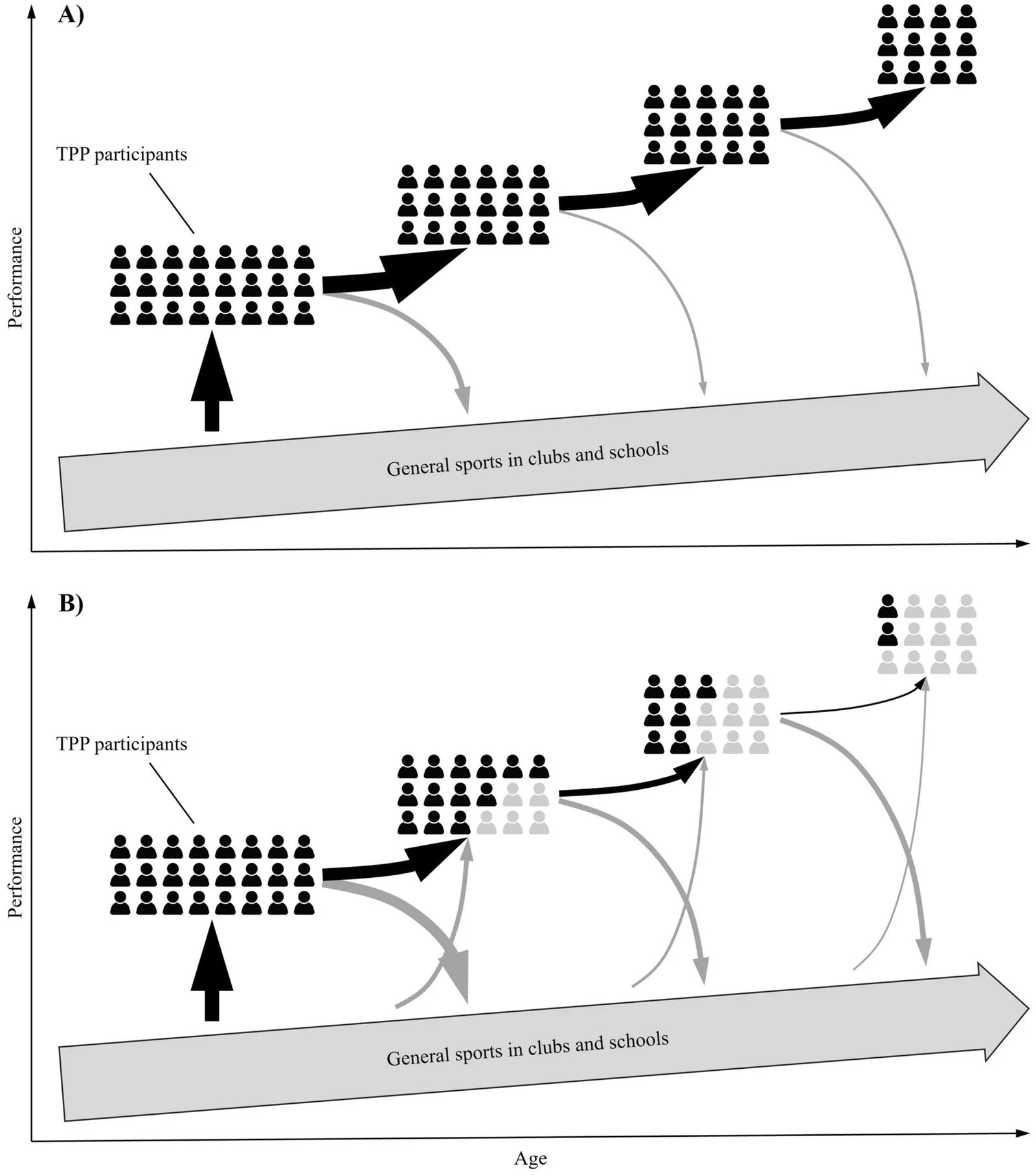
Illustration of two variants of recruitment of talent promotion programs (TPPs). **A** In each age category after a TPP’s initial age category, TPP participants are selected among TPP participants from previous age categories. There is little fluctuation among participants; the TPP population is very stable across age categories; and eventual successful senior athletes were already selected into the TPP’s initial age category and were then continuously involved in the TPP nurture through all age categories until peak performance. **B** In each age category after a TPP’s initial age category, TPP participants are selected from both previous TPP participants and non-participants. There is fluctuation within the TPP population—annual athlete turnover—and the TPP population is not stable across age categories. Eventual successful senior athletes may have been selected into the TPP’s initial age category or may have ‘side-entered’ the TPP at later ages. In consequence, early TPP participants and eventual successful senior athletes are partly different individuals. *Note.* Arrows and their width symbolize the flow of athletes

Based on these assumptions, it is expected that the TPP participants—who have the greatest potential and whose development is fostered through the TPP nurture—exhibit better performance progress over time and enlarge their lead in performance over non-participants across increasing age (see Fig. 1a). Athletes who are not selected for a TPP at a young age thus have reduced chances to be selected at a subsequent age, and their chances further decline across growing age. Therefore, after the initial TPP age category, participants in subsequent age categories are selected from participants in previous TPP age categories; the TPP population is highly stable over time; and consequently, eventual successful senior athletes have developed via early selection and then continuous TPP nurture through all age categories until senior peak performance (Fig. 1a).^3^

On the other hand, TPPs sometimes replace deselected athletes with new ‘side-entry’ athletes (athletes who enter a TPP after its initial age category [33]) (Fig. 1b). As a result, there is *athlete turnover* within the TPP population across age categories—fluctuation through exits and entries of athletes—and the TPP population is not stable over time. Eventual successful senior athletes may then have been in a TPP since its initial age category or may have ‘side-entered’ the program at later age categories. The successful senior athletes may then be partly different individuals than those involved in a TPP during young age categories (Fig. 1b).

The magnitude of annual athlete turnover is thus indicative of the *operating principle* of TPPs and the extent to which it corresponds to their central *idea*: in particular, the extent to which their operating principle is characterized by early talent identification and long-term continuous nurture, or by frequent selection, deselection, and replacement of youth athletes across age categories. The magnitude of the annual athlete turnover within TPPs may also provide an answer to another critical question: Have successful senior athletes developed through early selection and long-term continuous TPP nurture, or have they rather *emerged* via the course of recurrent procedures of athlete selection, deselection, and replacement across TPP age categories?

Indeed, empirical evidence indicates that the entry age of German soccer Bundesliga players into youth soccer academies, for example, is almost evenly distributed across the U10 to U19 age categories [63] (for corresponding examples, including other sports and countries, e.g., [1, 12, 18, 31, 33, 64–71]). Moreover, successful junior athletes (typically in their teens) and eventual successful senior athletes (typically in their 20s–30s) are not identical, but are two largely discrete populations over time [72, 73]: Most top junior athletes do not become top senior athletes, while most top senior athletes were not top junior athletes. One of the reasons is presumably that several predictors for early junior performance are the reverse of predictors of later senior performance (starting age, age to specialize exclusively in one’s main sport, practice amounts in one’s main sport and in other sports, and early performance progress [72, 74, 75]). This is also reflected in athletes’ age of entry into TPPs: higher junior performance is associated with younger entry into TPPs, whereas higher senior performance is associated with older entry into TPPs [1].

Correspondingly, studies have shown that TPPs deselect considerable numbers of participants and replace them with new ‘side-entry’ athletes across age categories (e.g., [19, 20, 33, 63, 66, 67, 76–79]). The annual athlete turnover varies from 17 to 81% in youth sport academies and from 15 to 81% in federations’ junior squads across studies and age categories [19, 80, 81]. The present meta-analysis aimed to provide robust and generalizable evidence on the magnitude of annual athlete turnover in Olympic TPPs. For this purpose, we synthesized the available empirical evidence from studies that quantified annual athlete turnover within youth sport academies and federations’ junior squads. We also investigated whether annual athlete turnover varies between youth sport academies and federations’ junior squads; across age categories; between sexes; and between older and recent studies.

## Methods

**Fig. 2:**
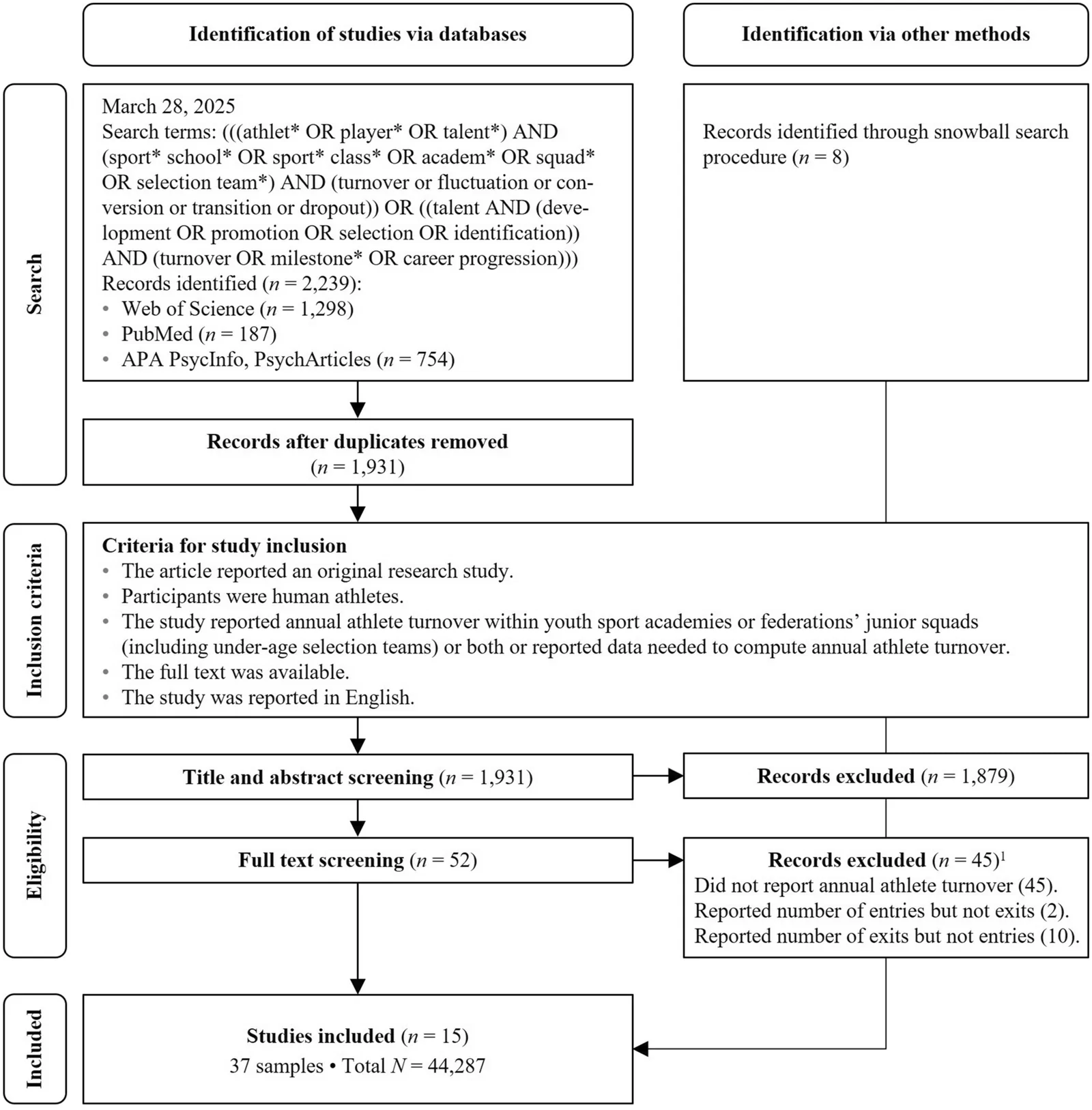
Flow diagram of the literature search and study coding. ^1^Sum > 45 because several studies were excluded by multiple criteria

The study search and selection procedure was guided by the PRISMA 2020 statement (Preferred Reporting Items for Systematic Reviews and Meta-Analyses [82]). We searched in the databases of Web of Science, PubMed, APA PsycINFO, APA PsycARTICLES, and Google Scholar on March 28, 2025, complemented by a snowball search process, for original studies that reported annual athlete turnover within youth sport academies or federations’ junior squads or both, or reported original data needed to compute annual athlete turnover. Figure 2 shows the flowchart of the major steps of the literature search and screening.

Study screening and selection, data extraction, and assessment of primary studies’ quality (see below) were independently done by both authors. The agreement was in each case 100%.

### Sample

The search yielded a total of 37 samples included in 15 study reports published from 2010 to 2025. Each study was coded for (1) descriptive data, (2) type of TPP (youth sport academies or federations’ junior squads), and (3) sample characteristics (country, sports, age category, and sex) (see Table S1 in the Electronic Supplementary Material, ESM). When a study reported various indicators of annual athlete turnover (e.g., from age 13 to 14 and 14 to 15 years), values were pooled for that study. Partly dependent samples were adjusted using Cheung and Chan’s method [83]

Table 1 shows characteristics of the sample. The total sample included 44,287 athletes; 26,079 (59%) were involved in youth sport academies and 18,208 (41%) in federations’ junior squads. Across studies, all sports of the Olympic Games were represented; 63% of the athletes were from team game sports (basketball, rugby, soccer); 36% were from multi-sport samples comprising all or several Olympic sports; and one study involved only alpine ski racers (1%). Eight studies (19 samples) were published from 2010 to 2017 and seven studies (18 samples) from 2018 to 2025.

**Table 1:** Sample characteristics and subsample sizes

(Sub) Sample	*k*^a^	*N*
Overall	37	44,287
Type of talent promotion program		
Youth sport academies	20	26,079
Federations’ junior squads	17	18,208
Ages^b^		
Under-12 years	6	2,987
12–16 years	15	17,942
17 + years	15	12,087
Sex		
Only female	6	4,042
Only male	27	24,157
Female and male	4	16,088

^a^ *k* number of samples, ^b^Sum of *k* < 37 and sum of *N* < 44,287 because one study did not report results separately by age categories

The athletes were from 42 countries^4^. The sex distribution was not reported for one study [12]; across all other studies, 17% of the participants were female and 83% male. Studies extended, on average, across 8.7 age years (sample-weighted mean; range 4 to 14 years). To compare annual athlete turnover across different age ranges, these were defined inductively based on the age categories divided within original studies: under-12, 12–16, and 17 years and older (Table 1).

### Meta-Analytic Procedure

All analyses were performed in the publicly available R environment. For each sample and every season-to-season transition, annual athlete turnover was computed by the formula of Güllich and Emrich [33, 63]:^5^

$$Annual\ athlete\ turnover = \frac{(number\ of\ entering\ athletes + number\ of\ exiting\ athletes)/2}{{total\ number\ of\ current\ athletes}}$$

Results are reported as the mean meta-analytic annual athlete turnover for the entire sample and then for defined subsamples. Studies were weighted by their inverse variance, computed via the “escalc” function of the metafor 4.6–0 package [84]. Random-effects models were estimated by restricted maximum likelihood estimator (REML) using the “rma” function of the metafor 4.6–0 package [84].

We followed Wang’s guidelines for meta-analyses of proportions [85] to quantify heterogeneity in the meta-analytic data and to identify potential outliers. The search for potential outliers included three steps: (1) the visual inspection of the Baujat plot [86] suggested that two studies had particular impact on both the pooled proportion and heterogeneity (see Figure S1 in the ESM). (2) We then screened for large externally studentized residuals. No study had a z-score >|3|, but the two studies indicated in step 1 had a z-score >|2|. (3) We therefore computed leave-one-out diagnostics for those two studies, respectively. Differences in the pooled proportions were negligible (see Table S2) and we decided to include all studies in our analysis.

The overall annual athlete turnover within TPPs was estimated by a random-effects model. Then, mixed-effects models with Wald’s *χ*^*2*^ were employed to analyze whether defined subsample characteristics moderated annual athlete turnover. For all moderator analyses, we followed the rule of thumb that *k* ≥ 5 is required for each subgroup [87] and tested for the following moderators: (1) type of TPP (youth sport academies vs federations’ junior squads); (2) age category (under-12, 12–16, and 17+ years); and (3) sex. In addition, to consider potential changes over time, (4) we also tested for recency of the evidence (studies divided by a median split into those published 2010–17 vs 2018–25). In all analyses, a value of *p* < 0.05 was considered as statistically significant.

The quality of the primary studies was assessed using the mixed-methods appraisal tool (MMAT [88]). The MMAT is an established tool for quality assessment of primary studies in systematic reviews and meta-analyses that provides a dedicated section for quantitative non-randomized studies, as included in this meta-analysis. To investigate potential publication bias, we inspected the funnel plot and computed Egger’s regression analysis.

## Results

The mean annual athlete turnover within TPPs is 36.3% (Table 2).^6^ It is slightly higher in federations’ junior squads than youth sport academies (Wald *χ*^*2*^ = 4.274, *p* = 0.039). The athlete turnover does not differ significantly across age categories (Wald *χ*^*2*^ = 1.308, *p* = 0.520), sexes (Wald *χ*^*2*^ = 0.902, *p* = 0.342), or between older and recent studies (Wald *χ*^*2*^ = 0.552, *p* = 0.458, Table 2).

**Table 2:** Meta-analytic mean annual athlete turnover in talent promotion programs (TPPs)

(Sub) sample	*M*^a^	95% CI^b^	*τ*^*2*^	*k*^c^	*p*
Overall	36.3%	32.7%, 39.8%	.011	37	<.001
Type of talent promotion program					
Youth sport academies	32.8%	28.1%, 37.6%	.010	20	<.001
Federations’ junior squads	39.9%	35.2%, 44.8%	.010	17	<.001
Age					
Under-12 years	32.0%	24.0%, 40.0%	.008	6	<.001
12–16 years	38.0%	33.2%, 42.7%	.008	15	<.001
17 + years	37.3%	30.9%, 43.8%	.015	15	<.001
Sex					
Female	32.1%	24.2%, 40.0%	.009	6	<.001
Male	37.1%	32.7%, 41.4%	.012	27	<.001
Female and male	37.1%	26.3%, 47.9%	.012	4	<.001
Recency of studies					
2010–2017	37.5%	33.0%, 42.1%	.010	19	<.001
2018–2025	34.6%	29.2%, 40.6%	.014	18	<.001

^a^*M* meta-analytic mean annual athlete turnover, ^b^ 95% *CI* lower and upper bound of 95% confidence interval (CI), ^c^ *k* number of samples

The dataset does not enable sport-specific comparisons. Yet, annual athlete turnover does not differ significantly between the largest sport-specific subsample, soccer, and the other Olympic sports (36.4%, 95% CI 32.3%–40.5%, vs 36.0%, 95% CI 28.9%–43.1%, Wald *χ*^*2*^ = 0.013, *p* = 0.911).

All primary studies had a high methodological quality and the risk of bias was generally low (see Table S3). The funnel plot shows a slightly skewed distribution and Egger’s regression was significant (Figure S3). Given the limited number of studies, the result should be interpreted with caution. If anything, it suggests that studies with small standard error and high athlete turnover within TPPs may have been less likely to be published.

## Discussion

The foundational idea of TPPs suggests that talents are identified and selected at young ages—often around puberty or younger—and are then involved in a long-term continuous TPP nurturing process until senior peak performance. This meta-analysis investigated the extent to which the operating principle of TPPs corresponds to their foundational idea. The central finding is that the factual operating principle of the TPP system is different than its original idea. In contrast to its central idea, the evidence suggests that the operating principle of the TPP system is characterized by significant annual athlete turnover, reflecting frequent selection, deselection, and replacement of youth athletes across age categories.

The meta-analysis included studies of athletes from all Olympic sports and 42 countries, and the evidence is robust across youth sport academies and federations’ junior squads, different ages and sexes, as well as older and recent studies. This suggests that the present evidence reflects a widespread, if not general, principle of TPPs. Furthermore, the meta-analytic evidence is consistent with studies showing that most squad careers only last 1–2 years [33, 63, 67]. It is also consistent with the meta-analytic evidence that several predictor effects on early junior performance and on later senior performance are opposite [74, 75], and early successful junior athletes and later successful senior athletes are two largely discrete populations over time [72, 73].

One might ask whether the athlete turnover primarily originates from deselection and less so from ‘side-entry’ of athletes across age categories. For example, the design of ‘pyramidal’ TPP systems implies deselection of athletes across increasing age. However, the notion is refuted by the evidence: Among the studies that reported annual athlete entries and exits separately (*k* = 33, *N* = 32,353; 73% of athletes in the total sample), average annual entries were 36.6% and exits 32.1% of yearly TPP athletes (under-12 years: 42.9% and 19.4%, 12–16 years: 41.3% and 31.7%, and 17+ years: 27.8% and 35.6%). Another question one might ask is whether the athlete turnover only takes place among a part of the TPP population while the other part persists in a TPP through the age categories. For example, the future successful senior athletes may remain unaffected by the athlete turnover, which only happens among other youth athletes. However, the notion is countered by empirical evidence in two regards: (1) numerous studies have shown wide dispersion of the TPP entry age of successful senior athletes, implying many ‘side-entries’ [12, 18, 31, 33, 63–70], where (2) higher-performing senior athletes entered TPPs years later than lower-performing peers [1]—the higher the senior performance level, the larger the proportion of late ‘side-entry’ athletes.

**Fig. 3:**
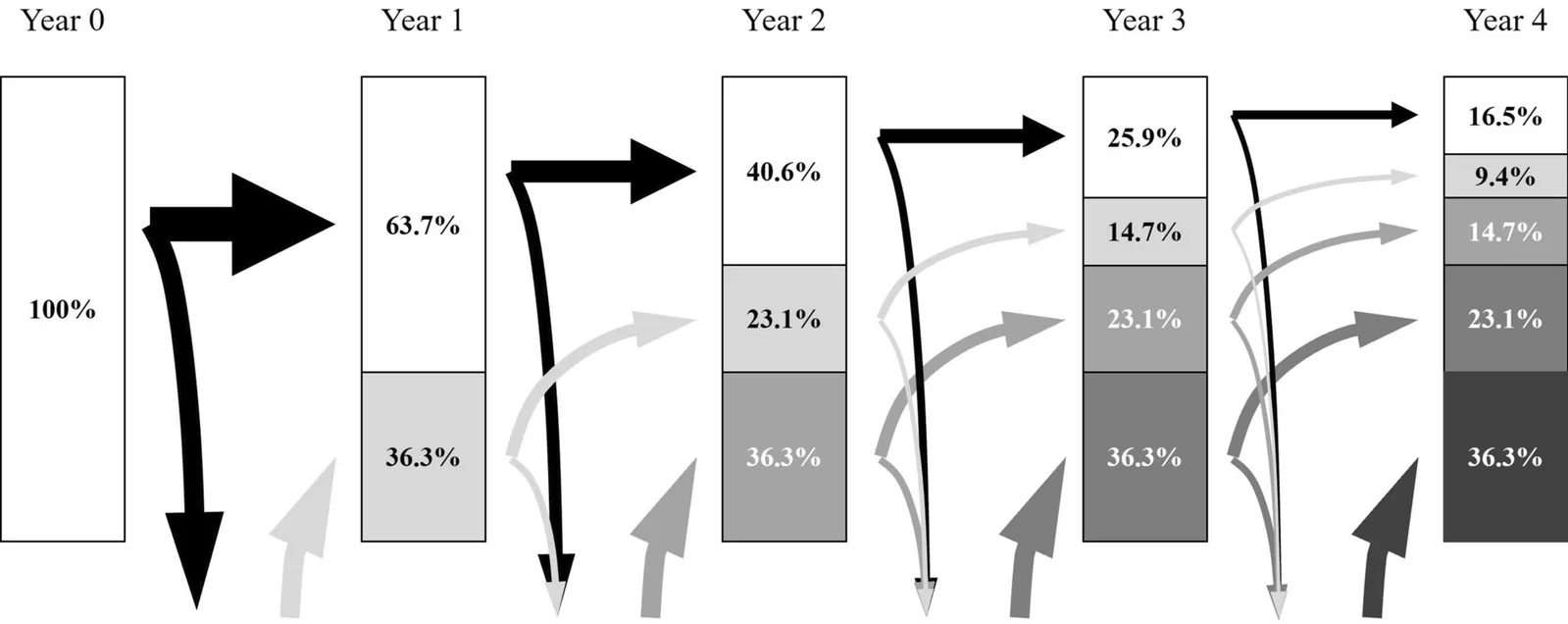
Illustration of the flow of athletes in talent promotion programs (TPPs) based on the empirical evidence. *Note.* Arrows and their width reflect annual athlete flow and its magnitude. Rectangles and their sizes reflect proportions within the TPP population in each year. Each cohort recruited in Years 0–4 has a different shade of grey of their arrows and rectangles (white to dark grey), illustrating how each recruited cohort diminishes across subsequent age categories. Within each year, percentages in retangles reflect the composition of the TPP population of athletes selected that respective year or 1, 2, 3, or 4 years before (distinguished by the shades of grey according to their year of recruitment). For example, the population in Year 2 includes 40.6% selected in Year 0, 23.1% in Year 1, and 36.3% selected in Year 2 itself. Of the population in Year 4, 16.5% were selected in Year 0, 9.4% in Year 1, 14.7% in Year 2, 23.1% in Year 3, and 36.3% in Year 4 itself. Total number of TPP participants held constant across age categories for clarity.

The distribution of lengths of squad careers reported in several studies [33, 63, 67] implies that the probability of deselection and replacement of TPP athletes is similar among those who have just been selected for a given season and those already involved in a TPP for several seasons. Considering this evidence, the mean annual athlete turnover of 36.3% implies that more than half of the TPP population is exchanged every two years. See Fig. 3 for an illustration.

Every season, many youth athletes who were previously ascribed the greatest potential and were involved in the TPP nurture are replaced with others who were previously ascribed inferior potential and did not participate in the TPP nurture, but who have developed more prosperously outside the TPP and are now ascribed greater potential. The finding implies that most talent-identified youth athletes subsequently develop worse than predicted by the talent identification procedures. At the same time, many others, including the majority of future successful senior athletes, are initially dismissed, but subsequently develop better than predicted by talent identification procedures. Accordingly, most talent identification and selection decisions are soon considered invalid and are revised.

Based on this evidence, the major operating principle of TPPs can be described as follows: TPPs try out many youth athletes and deselect most of them again soon to try out others. Correspondingly, rather than developing through early talent identification and long-term continuous TPP nurture, the collective of successful senior athletes *emerges* via the recurrent procedures of selecting, deselecting, and replacing youth athletes across TPP age categories. This also implies that most talents are rather identified a posteriori than a priori.

### Theoretical Considerations

The present meta-analysis complements a series of recent reviews and meta-analyses [1, 72–75, 89, 90] that empirically tested the validity of several fundamental assumptions of traditional giftedness, talent, and expertise theory [91–98]. The assumed premises (1) that successful juniors and later successful seniors are largely the same athletes over time; (2) that starting sport-specific practice at a younger age better facilitates long-term performance development; (3) that accumulating a larger amount of organized sport-specific practice leads to a higher level of eventual senior performance; (4) that childhood/adolescent non-organized peer-led play facilitates long-term performance development; (5) that accelerated childhood/adolescent performance progress leads to higher eventual senior performance; (6) that exceptional junior performance is a prerequisite for later exceptional senior performance; (7) that beginning TPP nurture at a younger age facilitates later senior performance; and (8) that predictors of junior performance also predict later senior performance, have all been revealed to be at odds with the empirical evidence. The present study adds that the assumed premise (9) that the operating principle of TPPs is characterized by early talent identification and long-term continuous nurture also is at odds with the empirical evidence.

TPPs principally consist of two components and their potential interplay: (1) The *identification and selection* of the (supposed) talents, and (2) their *‘processing’* through the TPP nurture in terms of environments and resources provided and interventions applied to the talent-identified youth athletes. The idea of TPPs implies the assumptions (a) that the talents with the greatest long-term potential are accurately identified and distinguished from peers with less potential within the ‘risk population’—the youth athletes considered for potential selection—at young ages, and (b) that the TPP nurture is superior to developmental conditions in general sport clubs and schools outside the TPP system in facilitating participants’ long-term performance development. However, the validity of the assumptions is uncertain, if not falsified. Higher or lower athlete turnover may therefore reflect varying accuracy of talent identification, varying efficacy of TPP nurture, or an interplay of both—a question that has not been systematically investigated to date.

Talent *identification* implies the assumption that individual differences in talent indicators at a young age predict individual differences in future long-term performance development and in eventual senior peak performance. Despite more than five decades of intensive relevant research [99, 100], the prognostic validity of available methods of talent identification has typically remained poor. As an example, Güllich and Larkin [17] reviewed the prognostic validity of talent indicators commonly used in talent identification procedures in youth soccer. Coach rating; players’ physique (height, weight, body composition); physical abilities (speed, agility, power, endurance, flexibility); perceptual-technical ball control skills (dribbling, passing, shooting, juggling); perceptual-tactical skills; and psychological characteristics (including motivation, commitment, confidence, volitional skills, coping skills, self-efficacy, and anxiety), assessed at a mean age of 12 years across studies, each explained on average 0–4% of the reliable variance of playing performance at a mean age of 17 years. When integrating the presumed talent indicators in multidimensional approaches, indicators at a mean age of 13 years explained on average 7% of the reliable performance variance at a mean age of 16 years (for consistent reviews, including other sports, see [44, 101–103]).

The reason for the poor prognostic validity is not that coaches, scouts, and staff are not doing their job well, but is rather rooted in the nature of the subject. There are multiple issues that impede valid estimation of youth athletes’ long-term potential and forecasts of individual differences in long-term performance development.

For example, first, athletes with different compositions of qualities (physical and physiological attributes, perceptual-technical and perceptual-tactical skills) can excel, and performance factors are to some extent mutually compensable.

Second, performance develops in an interplay of the task (e.g., physical abilities, technical and tactical skills, team interaction, opponents, competition rules), the person (e.g., biological maturation, health, motivation, commitment), and the environment (e.g., opportunities, practice, coaches, teammates, parental support, academic demands, chance) [39, 41, 104, 105]. Characteristics of the task, the person, and the environment (1) all differ inter-individually; (2) they change intra-individually over time; (3) different developmental factors may develop at different timing and rate intra-individually (heterochronicity of developmental components: for example, physical growth, psychological development, coaching, parental support, and interests within and outside of sports); and (4) the heterochronic intra-individual changes over time vary inter-individually. This also implies that an athlete’s long-term potential is not static but may change over time and is malleable.

Third, selecting the highest-performing youth athletes implies systematic selection bias in three regards: (1) many have an accelerated biological maturation (especially early onset of puberty and the growth spurt); (2) many have been born early within their age year (relative age effect, RAE); and (3) many have already had large amounts of sport-specific practice, with little or no practice in other sports, before the age of selection [6, 74, 75, 102, 106–112]. Each of these factors is associated with increased junior performance, but the effects diminish or are even reversed by adulthood [74, 75, 103, 111, 113–123]. Therefore, when accelerated early performance is (partly) based on these factors, performance progress often subsequently flattens, and the early performance advantage is not sustainable.

Fourth, in addition to the preceding, other predictor effects on youth performance and on later senior performance are also different or even opposite (main-sport starting age, participation in practice and competitions in other sports, early performance progress). Correspondingly, most top youth athletes do not become top senior athletes, while most top senior athletes were not top youth athletes, and top youth athletes and top senior athletes are largely two discrete populations [73, 89].

Fifth, tests of supposed talent indicators possess imperfect reliability.

Finally, coach assessment of youth athletes’ potential, in particular, may have poor objectivity (inter-rater reliability) [124, 125]. A youth athlete may be ascribed great potential by one coach or scout, but inferior potential by another.

These issues make it extremely difficult, if not impossible, to accurately distinguish youth athletes with greater and lower long-term potential within the risk population and to forecast individual differences in long-term senior peak performance.

The *processing* component in terms of the TPP nurture is based on the assumption that TPP involvement facilitates participants’ long-term performance development. Therefore, individual differences in participation in TPP nurture are supposed to predict individual differences in long-term performance development and eventual senior peak performance.

There are many cross-sectional qualitative and quantitative descriptions of program features and of participants’, coaches’, staff’s, and stakeholders’ subjective perception of TPPs [12, 13, 27, 28, 30, 32, 93, 126–132], often referring to the Holistic Ecological and the Talent Development Environment approaches [28, 133–135]. The results mostly reflect positive subjective evaluation of TPPs. However, a validation of the Holistic Ecological and Talent Development Environment approaches in terms of comparing athletes across the highest levels of senior performance with regard to their former childhood/adolescent environmental factors is not available to date.

Investigation of long-term performance-related effects of TPP nurture is generally limited. Where studied, multi-year longitudinal studies often failed to demonstrate a consistent positive effect of TPPs on participants’ performance development [3, 14, 15, 33]. TPP participants typically showed higher performance than controls at various time points; some TPP participants showed better performance progress over time than controls while others did not; and overall, multi-year performance progress was typically not better among TPP participants than controls.

Studies comparing athletes across the highest senior performance levels regarding TPP environments, resources, and interventions provided during their former childhood and adolescence are still lacking to date. Yet, the evidence that higher-performing senior athletes entered TPPs years later than lower-performing senior athletes [1] suggests that the developmental environments in their home sport clubs and schools alone, without TPP involvement, were favorable for their long-term development, if not superior to TPP environments.

The design of many TPP measures has a time-economic hard core (for the economy of time in general, see [136, 137]): Most features are aimed to expand the time for sport-specific practice and competitions (extensive time economy) and/or to use that time efficiently in terms of increased performance gain per invested time (intensive time economy; for the directionality of TPP features regarding extensive and intensive time economy, see [24, 33]). The assumed effect of practice-related extensive time economy has been challenged by evidence from the highest levels of senior performance [72, 74, 75]. In addition, the TPP nurture may also impact other major outcome dimensions than only athletic performance, including available time for other important developmental activities, academic performance, along with physical and psychosocial wellbeing and health. In this way, the TPP nurture may elicit unintended consequences by imposing increased (immaterial) costs and risks on the participants: Increased demands on time through the intense training and competition schedule, appointments with athlete service staff, and travel time; reduced time for academic pursuits, missed lessons and tests, constrained time with family, friends, and community, and constricted non-sport leisure activities; changing to a new school, relocating, and leaving one’s family; increased cumulative physical load along with compromised physical health and injuries; chronic time stress, performance pressure, declining academic performance, compromised psychosocial wellbeing, athletic and academic burnout; and also abuse of youth athletes [3, 14, 30, 138–143]. Children and adolescents may be particularly vulnerable to these factors. Detrimental effects may outweigh or exceed potential positive effects of the TPP participation, and for many youth athletes, the net effect of the TPP involvement may hamper rather than facilitate their development and may even lead to premature dropout.

TPPs’ personnel deal with the uncertain validity of fundamental premises underlying the central idea of TPPs by conducting a diverging operating principle in terms of trying out many youth athletes and expanding that number through sizeable annual athlete turnover. Although diverging from the original idea of TPPs, this operating principle may be functional by increasing the chances that future successful senior athletes are included in TPPs at some time and thereby minimizing the number of successful senior athletes who have developed outside the TPP system. The latter are antithetical to the societal confidence in the TPPs and thus compromise their legitimization function [33, 63, 144, 145].

At the same time, it may be functional for the stabilization of TPPs’ internal and external legitimization to uphold the rationalized myth of accurate early talent identification and effective long-term nurture (for the functionality of rationalized myths in organizations in general, see [146]). Relatedly, it may be functional for TPPs’ personnel and stakeholders to uncouple talk and actions to some extent (for the general functionality of uncoupling talk, decisions, and actions in organizations, see [147]). That is, it may be functional to display the operating principle of early talent identification and long-term continuous nurture, while the factual operating principle is mainly trying out many youth athletes and deselecting most of them again within a short time and trying out others.

### Practical Implications

The present data do not suggest direct practical implications: High or low annual athlete turnover is primarily a descriptive indicator of the operating principle of TPPs, rather than a goal variable to pursue. The question of whether higher or lower collective senior success of TPPs (i.e., the aggregated later successes of senior athletes previously involved in a TPP) is associated with higher or lower childhood/adolescent athlete turnover has not been systematically investigated. Furthermore, given that athlete turnover is apparently a relatively widespread, if not general, principle of TPPs, TPPs’ personnel and stakeholders can be confident that it is similar among competing TPPs and nations. Nevertheless, if people want to develop TPPs towards their original idea and reduce athlete turnover by improving the accuracy of talent identification and efficacy of TPP nurture, an integration of the present and recent evidence suggests several implications to consider.

The finding that higher senior performance is associated with older age of selection for TPPs and longer development outside TPPs [1] implies that particularly early TPP nurture is dispensable, if not detrimental, and suggests postponing selection to later ages. This may ideally be combined with systematic strengthening of talent development in general sport clubs and schools outside TPPs.

Concerning talent *identification and selection*, early junior performance alone—whether in competition or representative tests, evaluated by objective data or subjective coach assessment, or both—does not accurately reflect a youth athlete's long-term potential and is not a sensible selection criterion [73, 89]. Furthermore, selecting by early performance may have a dysfunctional ‘radiating’ effect: Youth athletes, their coaches, and parents are incentivized to reinforce early performance acceleration already before the selection age of TPPs. This is often pursued via starting sport-specific practice early, focusing on a single sport, and intensifying sport-specific practice, with little or no practice in other sports—the participation pattern associated with elite junior performance but not long-term senior top performance [74, 75].

Likewise, when evaluation of TPPs or their coaches and staff—which may be critical to funding and continuation—is based on junior performance or short-term progress, including progression to the next TPP age or stage, this may elicit a dysfunctional incentive structure. It is then individually rational for managers, coaches, and staff to select the most accelerated early performers and, once selected, further accelerate their short-term performance—which, however, often happens at the expense of participants’ long-term prospects. Alternatively, to consider sustainability, evaluations of TPPs along with their coaches and staff should assess participants’ development through subsequent years until senior peak performance.

Furthermore, identification of the youth athletes with the greatest long-term potential may consider early indicators beyond just top junior performance. The available evidence suggests that above-average, but not top, junior performance together with considerable, but not excessive, sport-specific practice, and multi-year practice and competitions in 2–3 sports are indicators of long-term potential [72, 74, 75].

Finally, when considering junior performance in talent identification and selection procedures, it appears plausible to apply several ‘corrective adjustments:’ i.e., to evaluate athletes’ youth performance relative to the timing of their biological maturation (especially onset of puberty and growth spurt); to their relative age within their age year; and to the amount of sport-specific practice accumulated before the selection age [6, 74, 75, 103, 106–112]. Yet, although plausible, an empirical validation of the approach in terms of more accurate identification of future senior top performers is still pending [148].

Concerning the ‘*processing*’ of the talent-identified youth athletes, many features of the TPP nurture aim at expanding and intensifying sport-specific practice, competitions, and athlete services. The increased supply of program resources to the TPP participants is often associated with intensified expenditure of their individual resources (time, their body, physical and psychosocial wellbeing, health, motivation). This may lead to unintended consequences including increased individual costs (especially opportunity costs—the lost benefit of foregone other activities) along with increased individual educational, wellbeing-, health-related, and motivational risks.

To date, there are no empirically substantiated benchmarks indicating at what point exactly increased demands of TPPs on their athletes turn into dysfunctional effects that are detrimental to their long-term performance development (or other outcomes concerning education, wellbeing, health, and motivation), and this may be highly individual and age-dependent. Yet, frequent negative outcomes for TPP participants [3, 14, 30, 138, 139–143] suggest that this point is exceeded in many cases. This is perhaps one of the reasons why senior world-class athletes, compared with lower-performing peers, entered TPPs at later ages, implying that the world-class athletes developed outside TPPs—unaffected by their potential dysfunctional effects—until older ages [1, 75].

It is therefore important to increase awareness that TPPs may elicit intended consequences, but also unintended consequences in terms of increased costs and risks imposed on youth athletes. To prevent potential dysfunctional effects, TPPs may increase awareness among coaches and staff and carefully monitor indicators of increasing individual opportunity costs along with risks to participants’ physical and psychosocial wellbeing and health, academic performance, motivation, and commitment, and establish relevant early alert systems.

### Methodological Considerations

The study had several strengths such as being the first meta-analysis to synthesize the available studies of annual athlete turnover in TPPs; a large sample including over 40,000 youth athletes from all Olympic sports and 42 countries; and a central finding that is robust across types of TPPs, ages, sexes, and older and recent studies. Nevertheless, the study did have limitations.

Male and Western European athletes were over-represented in original studies, and within federations’ junior squads, soccer was over-represented. In addition, we found no study of athlete turnover in TPPs in Paralympic and non-Olympic sports. Also, as in any review, bias of language and availability is possible.

Furthermore, frequent late ‘side-entry’ athletes in TPPs suggest that they had sufficient opportunities for practice and competitions outside TPPs, just based on their home sport club or school alone. It may be that athlete turnover within TPPs is lower in sports and countries with more limited opportunities for practice and competitions outside TPPs. For example, in sports with high asset specificity [149, 150]—i.e., sports that require specific, often expensive equipment and facilities (such as a bobsled run, ski jump, or velodrome)—and in countries with less developed general sport systems, there may be fewer sport clubs and schools outside TPPs that can provide adequate opportunities.

### Future Directions

Future research on athlete turnover within TPPs should seek to involve more female athletes and more athletes from Paralympic and non-Olympic sports, from countries other than Western Europe, and from federations’ junior squads in other sports than soccer. Furthermore, comparisons of athlete turnover between TPPs in individual and team sports, more and less popular sports, sports with higher and lower asset specificity, and countries with more and less developed sport systems warrant research attention. The present evidence also raises several questions for future research.

Annual athlete turnover, as an indicator of TPPs’ operating principle, raises the question of whether higher or lower athlete turnover during junior age categories is associated with higher or lower later collective senior success of TPP participants, and whether there is some optimal magnitude of youth athlete turnover to maximize collective senior success. Such investigations require documenting and tracking annual entries and exits within TPPs, computing annual athlete turnover (such as by the metric used in the present study), along with later collective senior success of TPP participants. Additionally, the present evidence raises the question of the extent to which higher or lower athlete turnover within TPPs results from better or poorer accuracy of talent identification or higher or lower efficacy of the TPP nurture or an interplay of both.

Furthermore, the emphasis on extensive research on talent identification—with widely consistent findings over more than five decades—is contrasted by limited research attention to the purpose talent identification is done for: the effects of participation in the TPPs for which talent-identified youth athletes are selected. Although the TPP nurture is the heart of these programs and despite extensive public and private investments in TPPs, the available empirical evidence to underpin evidence-based policies and practices of TPPs is limited. A central research question is: In what characteristics of TPP environments, resources, and interventions provided during childhood and adolescence did top senior athletes differ and to what extent from peers performing just below (e.g., world class vs national class, international medalists vs non-medalists)? This kind of investigation may at the same time offer a starting point for the validation of recent proposals such as the Holistic Ecological and Talent Development Environment approaches [28, 133, 135].

Next, TPPs may elicit positive or negative effects on athletic and other major outcomes (opportunity costs, academic pursuits, physical and psychosocial wellbeing and health, motivation, and commitment), calling for the investigation of further research questions. An initial goal is to quantify positive and negative outcomes under TPP participation: What magnitude of which positive and negative outcomes in each of the major outcome dimensions does TPP participation elicit?

Benefits and costs elicited by TPPs may have different subjective (material or immaterial) value for each athlete and may occur at different probabilities and frequencies (e.g., reaching national finals vs winning an international medal; prestige gained from varying athletic success; foregoing an assigned homework vs missing an important school exam; a bruise or skin scratch vs an ACL rupture or bone fracture). Furthermore, positive and negative TPP effects may vary across age and across short and long terms, where both benefits and costs may accumulate over time (e.g., athletic skill, prestige, opportunity costs, declining academic performance, physical load, injuries). The question concerning the magnitude of positive and negative outcomes elicited by participation in TPPs can thus be complemented by another question, i.e., what ratio of

$$(\sum_{k = i}^{n} benefit \times probability \times value)/(\sum_{k = i}^{n} cost \times probability \times value)$$

does TPP participation in the different ages elicit in each of the major outcome dimensions at short and long terms? This will enable determination of a net balance of the cumulative benefits and costs across the major outcome dimensions, while considering the sustainability of TPP effects.

TPPs are typically composed of multiple measures that may vary across TPPs and ages (e.g., high-quality facilities and equipment; number of coaches and training sessions; amounts of athlete services such as physiotherapy, nutritional counselling, psychological support, academic assistance, etc.). This further extends the research questions above: What amounts of participation in what TPP measures—individually and in combination—at different ages lead to what ratio of

$$(\sum_{k = i}^{n} benefit \times probability \times value)/(\sum_{k = i}^{n} cost \times probability \times value)$$

in each of the major outcome dimensions in the short and long terms?

Finally, the evidence that higher-performing senior athletes entered TPPs years later than lower-performing peers implies that many successful senior athletes developed based on their home sport club or school alone over longer periods. This raises the final two research questions: (1) What characterized the environments and resources in their home club or school? And (2) in what aspects and to what extent did the developmental conditions within their home clubs or schools differ from those of peers already participating in TPPs during their early years?

## Conclusion

The foundational idea of TPPs is to select the most promising talents at a young age and involve them in a long-term continuous nurturing process. The evidence synthesized in this meta-analysis suggests that the factual operating principle of TPPs is different than their original idea. The mean annual athlete turnover of TPPs is 36.3%, reflecting frequent entries and exits of youth athletes across age categories. Unlike their foundational idea, the predominant operating principle of TPPs can be described as follows: TPPs try out many youth athletes and expand that number through sizeable athlete turnover. Most talent-identified youth athletes are deselected again soon and replaced with others who are then tried out. Factors of varying athlete turnover in TPPs and potential associations of more or less athlete turnover with varying long-term success of TPP participants are subjects for future research.

## Supplementary Information

Supplementary Material 1.

Supplementary Material 2.

## Data Availability

The original data are freely available in the Electronic Supplementary Material, Table S1.

## Abbreviations

95% CI: 95% Confidence interval
ACL: Anterior cruciate ligament
APA: American Psychological Association
e.g.: Exempli gratia
ESM: Electronic supplementary material
etc.: Et cetera
i.e.: Id est
k: Number of samples
N: Total number of participants
PRISMA: Preferred Reporting Items for Systematic Reviews and Meta-Analyses
RAE: Relative age effect
REML: Restricted maximum likelihood estimator
TID: Talent identification
TPP(s): Talent promotion program(s)
vs: Versus

## Footnotes

1 Some have labelled these programs “talent development programs” (TDPs). We refrain from using this term because the development of talents takes place both within and outside these programs. In addition, the term may stimulate misinterpretation in terms of programs developing talents (as objects), rather than talents themselves developing (as subjects), with potential support from these programs. Furthermore, some have used “talent identification” (TID) programs as an umbrella term referring to all the different processes of searching for, detecting, identifying, selecting, and promoting talents. We suggest the central purpose of these programs is to promote the performance development of selected talents, where talent identification (TID) is *instrumental* to talent selection and talent selection is *instrumental* to talent promotion [1].

2 Youth sport academies have been labelled “elite sport schools,” “top sport schools,” “talent schools,” “elite sports classes,” “centers of excellence,” or “talent performance centers” in some countries and sports [3, 11–16].

3 Some TPPs have a ‘pyramidal’ structure with stepwise reduction of participants across increasing age, implying deselection of athletes across age categories. Deselection may include athletes withdrawing from the sport. Furthermore, some TPPs may initially begin recruiting locally and then expand their gathering ground to regional and national scope, which may imply ‘side-entries’ across age categories.

4 Argentina, Austria, Belarus, Belgium, Bosnia and Herzegovina, Brazil, Bulgaria, Chile, Croatia, Czech Republic, Denmark, Estonia, Finland, France, Georgia, Germany, Greece, Hungary, Iceland, Ireland, Israel, Italy, Latvia, Lithuania, Luxembourg, Macedonia, Montenegro, Netherlands, Poland, Portugal, Romania, Russia, Serbia, Slovakia, Slovenia, Spain, Sweden, Tunisia, Turkiye, Ukraine, United Kingdom, and United States of America.

5 Entering and exiting athletes do not include athletes who advance from one age category or stage to another within a TPP or athletes who change from one academy to another.

6 For the annual athlete turnover within each original study, see Table S1 and the forest plot, Figure S2.
