# Supplementary material for: Annual Athlete Turnover of 36% in Olympic Talent Promotion Programs: A Meta-analysis
Source: Sports Med Open. 2026 Jun 30;12:82. doi: 10.1186/s40798-026-01050-9 (PMC13319627; doi:10.1186/s40798-026-01050-9)
Supplement: Supplementary file 1 [file 40798_2026_1050_MOESM2_ESM.pdf]

Annual athlete turnover of 36% in Olympic talent promotion programs: a meta-analysis

Sports Medicine

Electronic Supplementary Material

Arne Güllich[(a)*], Michael Barth[(b)]

Running head: Athlete turnover in talent programs

[(a)] Department of Sports Science
RPTU Kaiserslautern
Erwin-Schrödinger-Straße 57
67663 Kaiserslautern
Germany

* Correspondence to guellich@sowi.uni-kl.de

ORCID: http://orcid.org/0000-0001-6911-2236

[(b)] Department of Sport Science
University of Innsbruck
Fürstenweg 185
6020 Innsbruck
Austria

ORCID: http://orcid.org/0000-0001-5333-3122

Table S1 Study characteristics and turnover

Study	Age	Sex	n_m	n_f	Age_span	Sport	Country	TPP	n	Annual tunover
Ford et al., 2020-s1	U12	m	2320		14	soccer	Int	academy	2320	0.313
Güllich, 2014a-s1	U12	m	197		10	soccer	GER	academy	197	0.223
Goto et al., 2019-s1	U12	m	35		8	soccer	GBR	academy	35	0.167
Patel et al 2020-s1	U12	m	94		11	soccer	GBR	academy	94	0.352
Wartenberg et al., 2014	U12	mf	148	90	4	multiple	GER	academy	238	0.395
Alder et al., 2024-s1	U12	f		103	11	soccer	GBR	academy	103	0.456
Goto et al., 2019-s2	12-16	m	29		8	soccer	GBR	academy	29	0.381
Bidaurrazaga et al., 2019	12-16	m	94		4	soccer	ESP	academy	94	0.267
Ford et al., 2020-s2	12-16	m	2900		14	soccer	Int	academy	2900	0.295
Güllich, 2014a-s2	12-16	m	684		10	soccer	GER	academy	684	0.251
Noon et al., 2020	12-16	m	76		6	soccer	GBR	academy	76	0.547
Patel et al 2020-s2	12-16	m	196		11	soccer	GBR	academy	196	0.349
Votteler & Höner, 2017-s1	12-16	m	3127		8	soccer	GER	academy	3127	0.291
Alder et al., 2024-s2	12-16	f		169	11	soccer	GBR	academy	169	0.317
Ford et al., 2020-s3	17+	m	1914		14	soccer	Int	academy	1914	0.252
Güllich, 2014a-s3	17+	m	539		10	soccer	GER	academy	539	0.276
Patel et al 2020-s3	17+	m	65		11	soccer	GBR	academy	65	0.682
Votteler & Höner, 2017-s2	17+	m	1956		8	soccer	GER	academy	1956	0.348
Alder et al., 2024-s3	17+	f		72	11	soccer	GBR	academy	72	0.319
Güllich & Emrich, 2010		mf			9	multiple	GER	academy	11271	0.213
Güllich, 2014a-s4	12-16	m	189		6	soccer	GER	squad	189	0.498
Schroepf & Lames, 2017-s1	12-16	m	232		6	soccer	GER	squad	232	0.340
Votteler & Höner, 2017-s3	12-16	m	650		8	soccer	GER	squad	650	0.411
Votteler & Höner, 2017-s5	12-16	m	197		8	soccer	GER	squad	197	0.517
Güllich & Emich, 2012-s1	12-16	mf	2244	948	7	multiple	GER	squad	3192	0.440
Kalén et al., 2021-s1	12-16	m	3573		6	basketball	Int	squad	3573	0.475
Kalén et al., 2021-s3	12-16	f		2634	6	basketball	Int	squad	2634	0.356
Güllich, 2014a-s5	17+	m	870		6	soccer	GER	squad	870	0.395
Schroepf & Lames, 2017-s2	17+	m	404		6	soccer	GER	squad	404	0.478
Votteler & Höner, 2017-s4	17+	m	970		8	soccer	GER	squad	970	0.370
Votteler & Höner, 2017-s6	17+	m	226		8	soccer	GER	squad	226	0.448
Güllich & Emich, 2012-s2	17+	mf	902	485	7	multiple	GER	squad	1387	0.440
Kalén et al., 2021-s2	17+	m	1986		6	basketball	Int	squad	1986	0.223
Kalén et al., 2021-s4	17+	f		852	6	basketball	Int	squad	852	0.162
Barth et al., 2018-s1	17+	f		212	5	alpine skiing	AUT	squad	212	0.343
Barth et al., 2018-s2	17+	m	238		5	alpine skiing	AUT	squad	238	0.374
Velentza, 2017	17+	m	396		6	rugby	GBR	squad	396	0.546

m = male
f = female
mf = male and female

Int = international

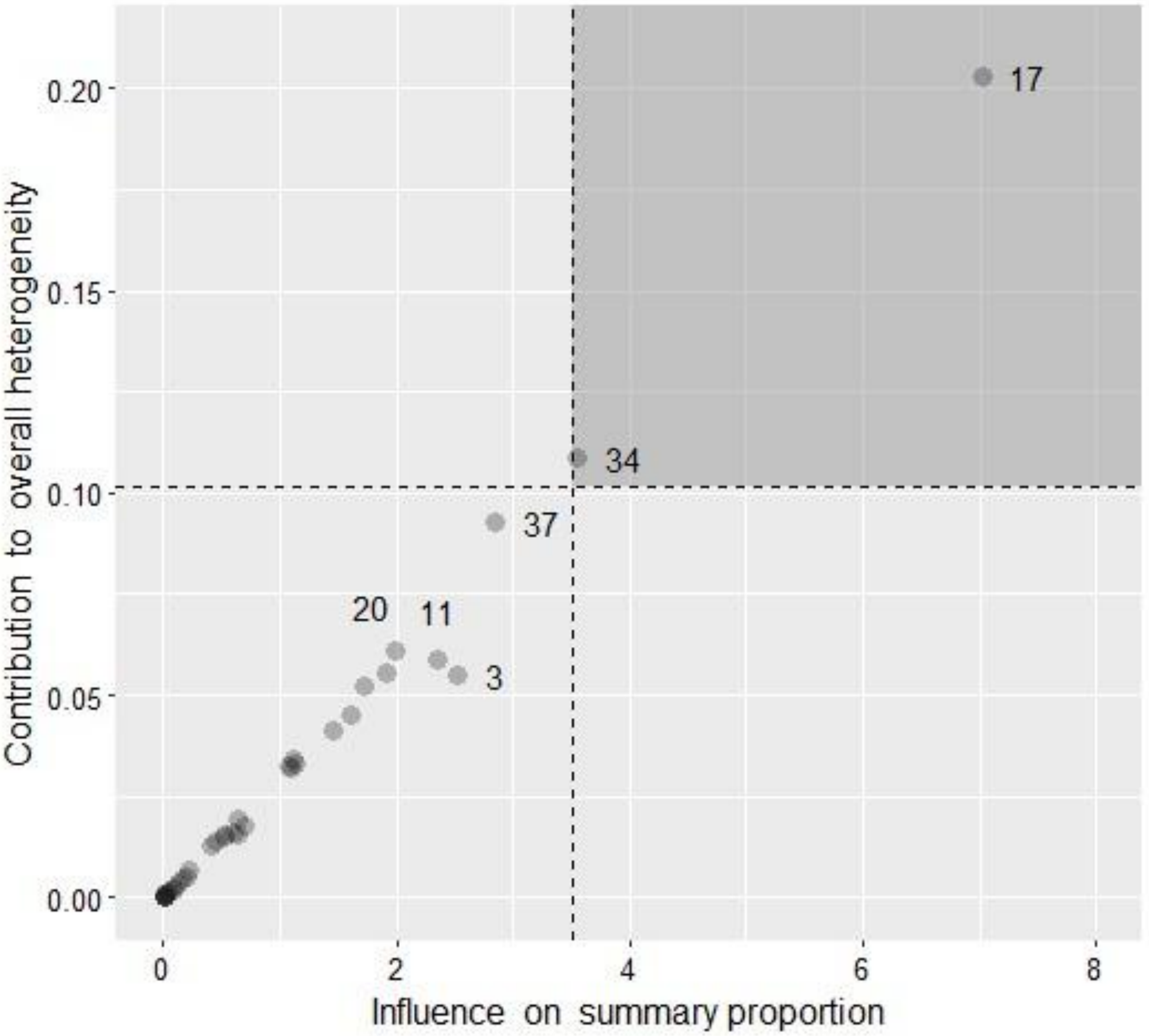

Fig. S1 Baujat plot.

Table S2 Leave-one-out diagnostics.

(Sub) Sample	*M*[a]	95% CI[b]	τ^2
Overall	36.3%	32.7%,39.8%	.011
Overall, leave out Patel et al., 2020-s3	35.5%	32.2%,38.8%	.009
Overall, leave out Kalén et al., 2021-s4	36.8%	33.4%,40.3%	.010

Note. [a] M = meta-analytic mean annual athlete turnover. [b] 95% CI = 95% confidence interval lower and upper bound.

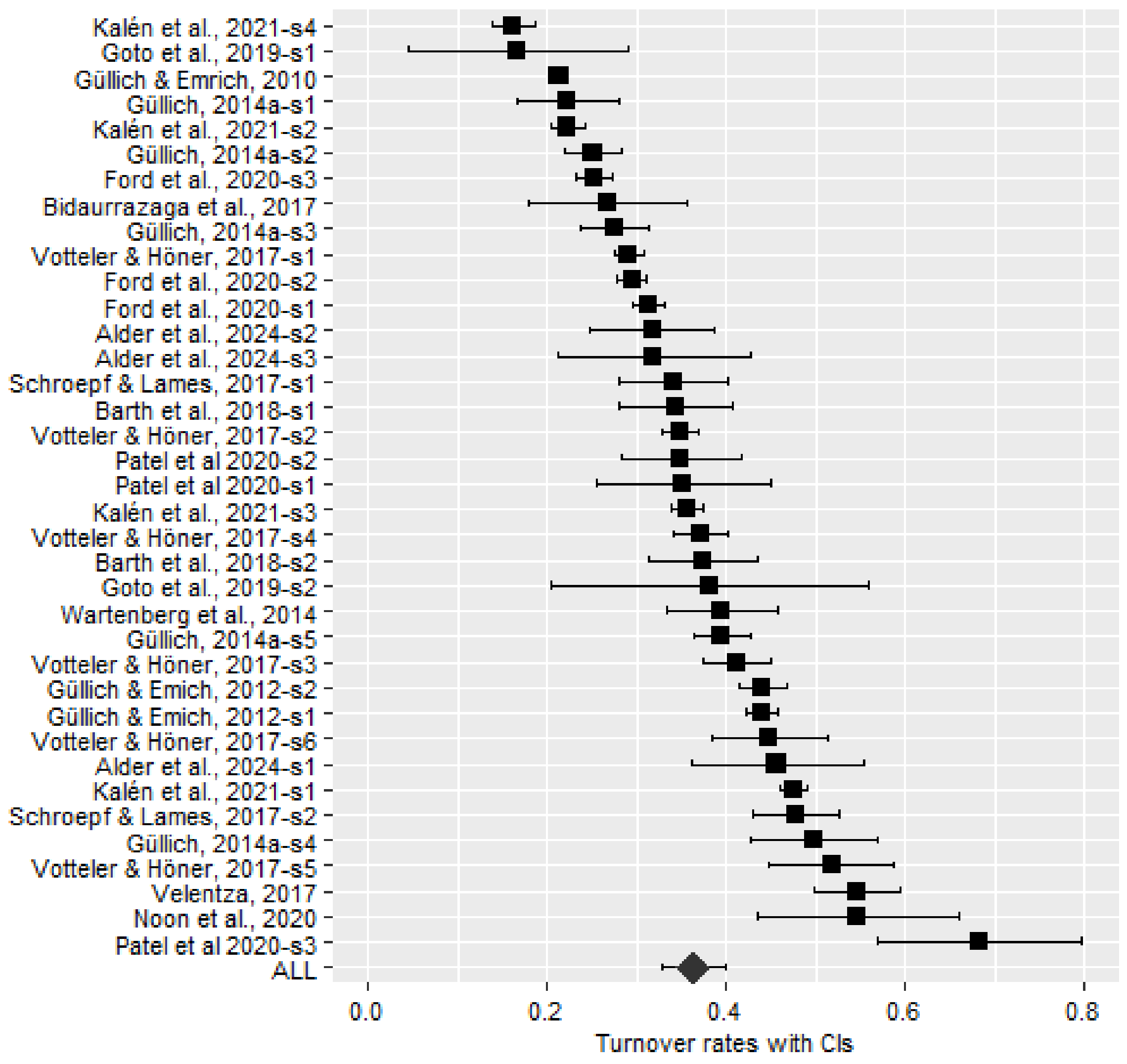

Fig. S2 Forest plot.
Note. The diamond represents the overall meta-analytic mean annual athlete turnover. Squares represent annual athlete turnover of each study. Error bars represent 95% confidence intervals.

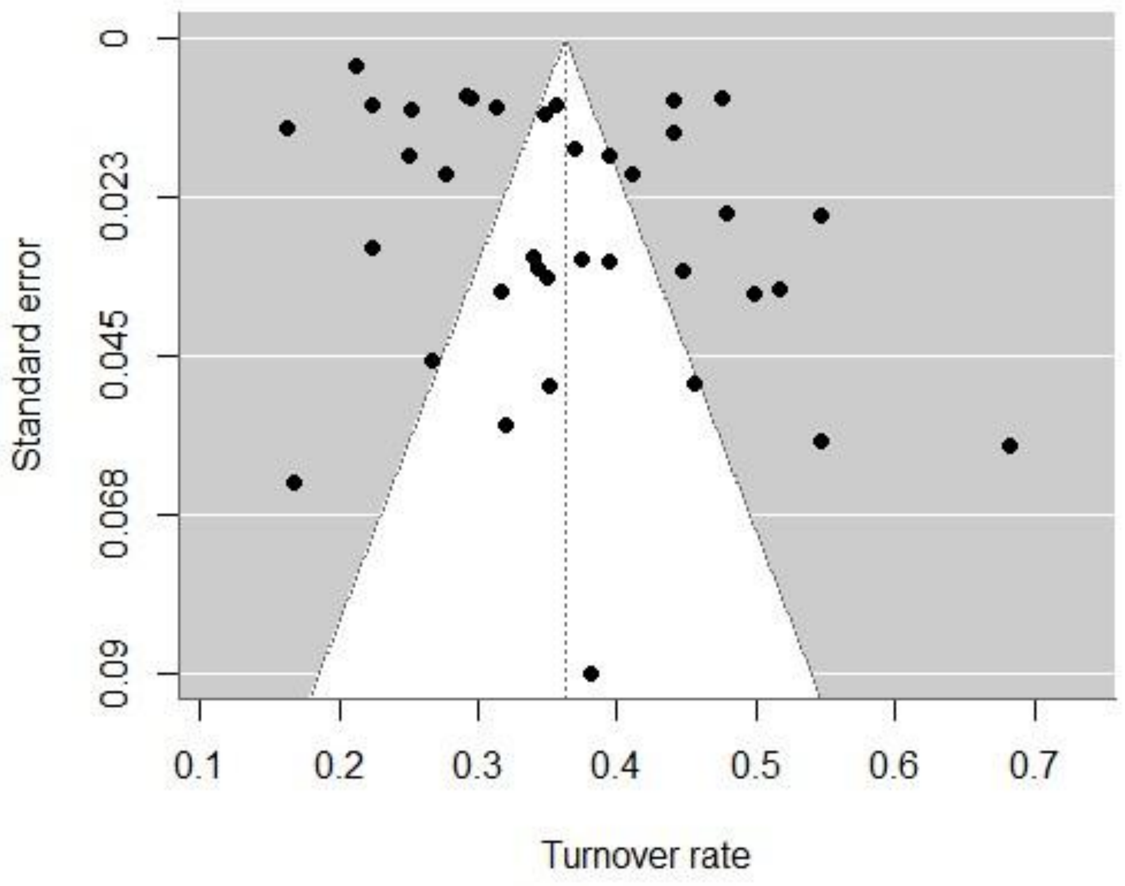

Fig. S3 Funnel plot. Egger's regression: b = 0.251 (0.204, 0.298), p = .008.

Table S3. MMAT[1] assessment.

Study	S1	S2	Q1	Q2	Q3	Q4	Q5	Score
Alder et al., 2025	Yes	Yes	Yes	Yes	Yes	Yes	Yes	100%
Barth et al., 2018	Yes	Yes	Yes	Yes	Yes	Yes	Yes	100%
Bidaurrazaga et al., 2019	Yes	Yes	Yes	Yes	Yes	Yes	Yes	100%
Ford et al., 2020	Yes	Yes	Yes	Yes	Yes	Yes	Yes	100%
Goto et al., 2019	Yes	Yes	Yes	Yes	Yes	Yes	Yes	100%
Güllich & Emrich, 2010	Yes	Yes	Yes	Yes	Yes	Yes	Yes	100%
Güllich & Emrich, 2012	Yes	Yes	Yes	Yes	Yes	Yes	Yes	100%
Güllich, 2014	Yes	Yes	Yes	Yes	Yes	Yes	Yes	100%
Kalén et al., 2021	Yes	Yes	Yes	Yes	Yes	Yes	Yes	100%
Noon et al., 2020	Yes	Yes	Yes	Yes	Yes	Yes	Yes	100%
Patel et al 2020	Yes	Yes	Yes	Yes	Yes	Yes	Yes	100%
Schroepf & Lames, 2017	Yes	Yes	Yes	Yes	Yes	Yes	Yes	100%
Velentza, 2017	Yes	Yes	Yes	Yes	Yes	Yes	Yes	100%
Votteler & Höner, 2017	Yes	Yes	Yes	Yes	Yes	Yes	Yes	100%
Wartenberg et al., 2014	Yes	Yes	Yes	Yes	Yes	Yes	Yes	100%

S1: Are there clear research questions? S2: Do the collected data allow to address the research questions? Q1: Is the sampling strategy relevant to address the research question? Q2: Is the sample representative of the target population? Q3: Are the measurements appropriate? Q4: Is the risk of nonresponse bias low? Q5: Is the statistical analysis appropriate to answer the research question?

[1] Hong QN, Pluye P, Fàbregues S, Bartlett G, Boardman F, Cargo M, et al. Mixed methods appraisal tool (MMAT). Version 2018. User guide. Montréal; 2018.

References in Table S1

Alder SE, Causer J, Champ FM, McRobert AP, Datson N, Andrew M. Talent identification and development processes of female soccer academies from the top three tiers in England. Journal of Expertise. 2024;7:130–48.

Barth M, Güllich A, Emrich E. The rich get richer and the poor get poorer - the Matthew mechanism as an approach to explain selection effects and the occurrence of multiple medalists in the “production” of international success in alpine ski racing. CISS. 2018;3:8. doi:10.15203/CISS_2018.008.

Bidaurrazaga-Letona I, Lekue JA, Amado M, Gil SM. Progression in youth soccer: selection and identification in youth soccer players aged 13-15 years. J Strength Cond Res. 2019;33:2548–58. doi:10.1519/JSC.0000000000001924.

Ford PR, Bordonau JLD, Bonanno D, Tavares J, Groenendijk C, Fink C, et al. A survey of talent identification and development processes in the youth academies of professional soccer clubs from around the world. J Sports Sci. 2020;38:1269–78. doi:10.1080/02640414.2020.1752440.

Güllich A. Selection, de-selection and progression in German football talent promotion. Eur J Sport Sci. 2014;14:530–7. doi:10.1080/17461391.2013.858371.

Güllich A, Emrich E. Elite sport and education in Europe. Brussels: European Commission; 2010.

Güllich A, Emrich E. Individualistic and collectivistic approach in athlete support programmes in the German high-performance sport system. European Journal for Sport and Society. 2012;9:243–68. doi:10.1080/16138171.2012.11687900.

Goto H, Morris JG, Nevill ME. Influence of biological maturity on the match performance of 8- to 16-year-old, elite, male, youth soccer players. J Strength Cond Res. 2019;33:3078–84. doi:10.1519/JSC.0000000000002510.

Kalén A, Lundkvist E, Ivarsson A, Rey E, Pérez-Ferreirós A. The influence of initial selection age, relative age effect and country long-term performance on the re-selection process in European basketball youth national teams. J Sports Sci. 2021;39:388–94. doi:10.1080/02640414.2020.1823109.

Patel R, Nevill A, Smith T, Cloak R, Wyon M. The influence of birth quartile, maturation, anthropometry and physical performances on player retention: observations from an elite football academy. Int J Sports Sci Coach. 2020;15:121–34. doi:10.1177/1747954120906507.

Noon MR, Eyre ELJ, Ellis M, Myers TD, Morris RO, Mundy PD, et al. The influence of recruitment age and anthropometric and physical characteristics on the development pathway of English academy football players. Int J Sports Physiol Perf. 2021;16: 199–207. doi:10.1123/ijspp.2019-0534

Schroepf B, Lames M. Career patterns in German football youth national teams – a longitudinal study. Int J Sports Sci Coach. 2018;13:405–14. doi:10.1177/1747954117729368.

Velentza E. A retrospective analysis of talent selection and progression within England’s rugby football union elite player performance pathway [Doctoral theses]. Chester: University of Chester; 2017.

Votteler A, Höner O. Cross-sectional and longitudinal analyses of the relative age effect in German youth football. Ger J Exerc Sport Res. 2017;47:194–204. doi:10.1007/s12662-017-0457-0.

Wartenberg J, Borchert T, Brand R. A longitudinal assessment of adolescent student-athletes’ school performance. Sportwissenschaft. 2014;44:78–85. doi:10.1007/s12662-014-0331-2.
